# Recent Advances and Prospects in RNA Drug Development

**DOI:** 10.3390/ijms252212284

**Published:** 2024-11-15

**Authors:** Hidenori Tani

**Affiliations:** Department of Health Pharmacy, Yokohama University of Pharmacy, 601 Matano, Totsuka, Yokohama 245-0066, Japan; hidenori.tani@yok.hamayaku.ac.jp

**Keywords:** RNA therapeutics, antisense oligonucleotides, small interfering RNAs, microRNAs, messenger RNAs, aptamers, CRISPR-Cas9 guide RNAs

## Abstract

RNA therapeutics have undergone remarkable evolution since their inception in the late 1970s, revolutionizing medicine by offering new possibilities for treating previously intractable diseases. The field encompasses various modalities, including antisense oligonucleotides (ASOs), small interfering RNAs (siRNAs), microRNAs (miRNAs), and messenger RNAs (mRNAs), each with unique mechanisms and applications. The foundation was laid in 1978 with the discovery that synthetic oligonucleotides could inhibit viral replication, followed by pivotal developments such as RNA interference’s discovery in 1998. The COVID-19 pandemic marked a crucial turning point, demonstrating the potential of mRNA vaccines and accelerating interest in RNA-based approaches. However, significant challenges remain, including stability issues, delivery to target tissues, potential off-target effects, and immunogenicity concerns. Recent advancements in chemical modifications, delivery systems, and the integration of AI technologies are addressing these challenges. The field has seen notable successes, such as approved treatments for spinal muscular atrophy and hereditary transthyretin-mediated amyloidosis. Looking ahead, RNA therapeutics show promise for personalized medicine approaches, particularly in treating genetic disorders and cancer. The continued evolution of this field, driven by technological innovations and deeper understanding of RNA biology, suggests a transformative impact on future medical treatments. The purpose of this review is to provide a comprehensive overview of the evolution, current state, and prospects of RNA therapeutics.

## 1. Introduction

### 1.1. Evolution of RNA Therapeutics

**Figure 1:**
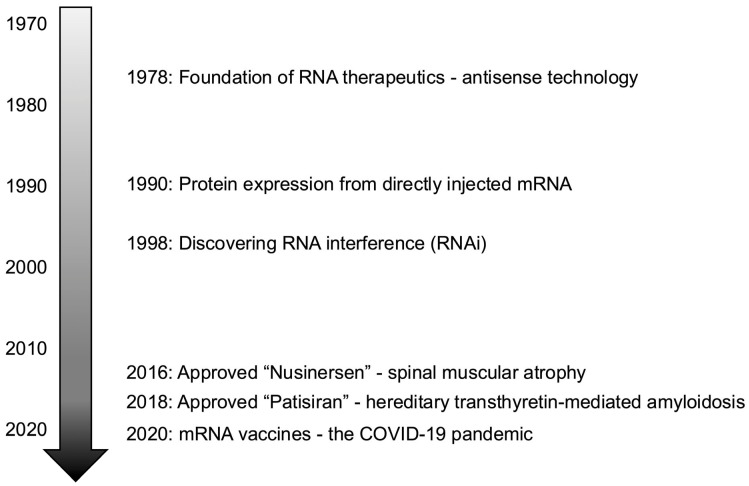
A comprehensive timeline illustrating key milestones in RNA therapeutics from 1978 to 2020. This figure highlights significant discoveries, technological advancements, and regulatory approvals that have shaped the field, culminating in the development of mRNA vaccines for COVID-19 in 2020.

RNA therapeutics has undergone a remarkable transformation since its inception in the late 1970s, revolutionizing the field of medicine and opening new possibilities for treating previously intractable diseases (Figure 1). The foundation of antisense oligonucleotide (ASO) technology was laid in 1978 with the discovery that synthetic oligonucleotides could inhibit viral replication [1]. This groundbreaking research demonstrated the potential to control specific gene expression, paving the way for RNA-based therapeutics. Subsequently, in 1990, a pivotal study reported that direct injection of messenger RNA (mRNA) into mouse muscle could induce protein expression [2], foreshadowing the potential of mRNA-based therapies and expanding the horizons of RNA therapeutics. One of the most significant turning points in the history of RNA therapeutics was the discovery of RNA interference (RNAi) by Andrew Fire and Craig Mello in 1998 [3]. This discovery greatly expanded the possibilities of gene silencing and led to their Nobel Prize in Physiology or Medicine in 2006. RNAi, with its powerful mechanism to degrade specific mRNAs and suppress gene expression, revolutionized RNA therapeutics. However, the path to clinical success in RNA therapeutics was not without its challenges. Fomivirsen (brand name Vitravene), the first RNA drug approved by the U.S. Food and Drug Administration (FDA) and by the European Medicines Agency (EMA) in 1998 and 1999, respectively, was expected to treat cytomegalovirus retinitis but faced economic difficulties due to poor sales, leading to the withdrawal of its marketing authorization in the EU in 2002 and in the US in 2006 by Novartis. This experience highlighted the challenges in delivery systems and stability of RNA drugs, emphasizing the need to address these issues for future success. The field of RNA therapeutics experienced a resurgence in the 2010s. The FDA and the EMA approval of nusinersen (Spinraza) for spinal muscular atrophy in 2016 and the FDA and the EMA approval of patisiran (Onpattro) for hereditary transthyretin-mediated amyloidosis in 2018 demonstrated the clinical efficacy of RNA therapeutics and accelerated research in this field [4]. The COVID-19 pandemic in the early 2020s marked a crucial turning point for RNA therapeutics. The rapid development and remarkable efficacy of mRNA vaccines showcased the potential of RNA technology to the world [5]. This success greatly accelerated interest in RNA-based approaches across the pharmaceutical industry, ushering in a new era of RNA therapeutics. Katalin Karikó and Drew Weissman, who established the base technology for mRNA vaccines, were awarded the Nobel Prize in Physiology or Medicine in 2023. Recent research has further expanded the scope of RNA therapeutics. For instance, the development of new genome editing approaches combining CRISPR–Cas9 gene editing technology with RNAi techniques is progressing. This is expected to enable more precise gene repair and control of complex gene networks. Additionally, the development of new RNA delivery systems utilizing nanotechnology is advancing, making efficient delivery of RNA drugs to specific tissues and cells increasingly possible. Furthermore, optimization of RNA design using artificial intelligence (AI), including machine learning technologies, is progressing. This is expected to accelerate the development of effective RNA drugs with fewer side effects. For example, it is becoming possible to predict the higher-order structure and stability of RNA sequences using AI to design optimal RNA drugs. The evolution of RNA therapeutics has been driven by close collaboration between basic research and clinical applications. It is expected that RNA therapeutics will continue to develop through the parallel advancement of new technology development and improvement of existing technologies. Applications in personalized medicine and rare disease treatment are anticipated, suggesting that RNA therapeutics has the potential to significantly change the future of medicine.

### 1.2. Versatility and Potential of RNA Therapeutics

RNA therapeutics represent a groundbreaking advancement in the field of medicine, offering unprecedented precision and specificity in targeting molecules and pathways previously deemed “undruggable” by conventional pharmaceuticals and antibody-based therapies. Conventional molecular modeling techniques, particularly molecular docking [6] and molecular dynamics simulations [7], play a crucial role in the development and understanding of RNA therapeutics. These methods provide valuable insights into RNA structure, dynamics, and interactions with potential drug molecules. This innovative approach to drug development has opened new avenues for treating a wide range of diseases that were once considered intractable. The versatility of RNA therapeutics is exemplified by the diverse mechanisms of action they employ. One such mechanism is “mRNA replacement therapy”, which involves the direct introduction of mRNA to compensate for dysfunctional proteins. This technique has shown promise in the treatment of genetic disorders and metabolic syndromes, where it can effectively replace non-functional proteins with their fully operational counterparts. Another powerful approach utilizes RNAi for gene silencing, enabling the suppression of specific gene expressions. This method has found applications in the treatment of diseases characterized by overexpression of certain genes, such as cancer and autoimmune disorders. Additionally, RNA therapeutics can modify RNA splicing patterns, either preventing the production of abnormal proteins or promoting the synthesis of beneficial ones. This technology offers new possibilities for treating genetic disorders caused by RNA splicing abnormalities. The remarkable aspect of these diverse approaches is that they all stem from a single RNA platform, allowing for rapid development and adaptability in RNA drug discovery. Consequently, RNA therapeutics can be designed and manufactured in a significantly shorter timeframe compared to traditional pharmaceuticals [8,9,10]. This agility was dramatically demonstrated during the COVID-19 pandemic, with the unprecedented speed of mRNA vaccine development. The fact that mRNA vaccines were developed and approved for clinical use within just one year of the pandemic’s onset serves as a powerful testament to the potential of RNA therapeutics [5]. However, these treatments are expensive, and lower costs are desired in the future. Furthermore, RNA therapeutics hold immense promise for the realization of personalized medicine. This approach enables the rapid development of customized treatments based on individual patients’ genetic profiles. This characteristic is particularly beneficial in the treatment of rare genetic disorders, offering new hope for many conditions that have previously lacked effective treatments or had no treatment options at all [11]. In conclusion, the diversity of RNA therapeutics symbolizes the innovative outcomes resulting from the convergence of medicine and life sciences. As a core technology poised to shape the future of healthcare, RNA therapeutics represent an exciting field with tremendous potential for further development and application in addressing complex medical challenges.

This review provides a comprehensive analysis of RNA therapeutics, covering its historical evolution from the 1970s to the present and highlighting key milestones. It explores the versatility and potential of RNA therapeutics as a platform technology, particularly in targeting previously “undruggable” molecules and its applications in personalized medicine. The review also examines current challenges in RNA therapeutics development, focusing on delivery systems, RNA stability, and off-target effects, while discussing innovative solutions and future directions. The goal is to demonstrate how RNA therapeutics are transforming modern medicine and their potential to revolutionize future healthcare approaches.

## 2. Types of RNA Therapeutics

### 2.1. Antisense Oligonucleotides (ASOs)

Antisense oligonucleotides (ASOs) represent a significant advancement in RNA therapeutics, offering a sophisticated approach to precisely modulate gene expression [12]. These short, synthetic single-stranded DNA or RNA molecules are meticulously designed to bind to complementary sequences in target RNAs, thereby influencing various aspects of RNA function and metabolism. The mechanisms of action of ASOs are multifaceted and depend on their specific design and chemical modifications [13]. One of the primary mechanisms by which ASOs exert their effects is through interaction with ribonuclease H (RNase H), an endogenous enzyme that cleaves the RNA strand in RNA–DNA hybrid regions. ASOs designed to leverage this interaction typically consist of a central DNA region flanked by chemically modified nucleotides, a structure known as a “gapmer” [14]. This configuration enables the ASO to effectively engage RNase H and promote targeted RNA degradation. Another crucial mechanism of ASO action involves the modulation of RNA splicing. By targeting specific RNA sequences involved in splicing, such as splice sites or regulatory elements, ASOs can alter the splicing patterns of target genes. This approach has proven particularly effective in treating genetic disorders caused by splicing defects, with nusinersen (Spinraza), an FDA-approved drug for spinal muscular atrophy, serving as a prime example of this therapeutic strategy. ASOs can also function as steric blockers, physically impeding the binding of various cellular machinery involved in RNA processing. This mechanism can be harnessed to inhibit translation initiation, regulate polyadenylation, or interfere with miRNA function, offering a versatile toolkit for manipulating gene expression at multiple levels [15]. The design and optimization of ASOs are increasingly benefiting from the application of AI techniques. These advanced computational approaches are expected to accelerate the development of more effective ASOs with reduced side effects. Furthermore, innovative strategies combining ASOs with CRISPR–Cas9 systems are being explored, potentially enabling more precise gene repair methodologies. Delivery systems for ASOs continue to evolve, with ongoing research focusing on the development of nanoparticles targeting cell-specific receptors and exosome-based delivery systems. These advancements promise to enhance the efficiency of ASO administration and expand their therapeutic potential [16]. The unique ability of ASOs to modulate gene expression offers new therapeutic avenues for diseases that have been challenging to address with conventional pharmaceuticals. Particularly in the realm of rare genetic disorders and neurodegenerative diseases, ASOs hold great promise. As research and development in this field progress, it is anticipated that ASOs will continue to evolve, establishing themselves as effective therapeutic modalities for an increasingly broad spectrum of diseases.

### 2.2. Small Interfering RNAs (siRNAs)

RNA interference (RNAi) mediated by small interfering RNAs (siRNAs) represents a potent mechanism for gene expression suppression [17]. These double-stranded RNA molecules, typically comprising 20–25 base pairs, possess the remarkable ability to degrade mRNA post-transcriptionally, thereby effectively silencing specific genes with high precision and efficiency. The process of gene silencing via siRNA commences with the introduction of double-stranded RNA into the cellular environment. This double-stranded RNA is subsequently processed by an enzyme called Dicer, which cleaves it into shorter siRNA molecules. These newly generated siRNAs are then incorporated into the RNA-induced silencing complex (RISC). Once integrated into RISC, the siRNA utilizes its sequence specificity to recognize and bind to the target mRNA. This binding event triggers the Argonaute 2 (Ago2) protein, a key component of RISC, to cleave the target mRNA. The cleaved mRNA fragments are rapidly degraded by cellular machinery, ultimately resulting in the suppression of gene expression. It is worth noting that this process exhibits an extraordinarily high level of specificity, to the extent that even a single base mismatch in the siRNA sequence can significantly impact target recognition and silencing efficiency. The therapeutic potential of siRNA-based drugs has been demonstrated across a wide spectrum of diseases, with several siRNA-based pharmaceuticals already available in the market. For instance, patisiran (Onpattro) has been approved for the treatment of hereditary transthyretin-mediated amyloidosis [18], givosiran (Givlaari) for acute hepatic porphyria, and inclisiran (Leqvio) for reducing low-density lipoprotein (LDL) cholesterol levels in patients with atherosclerotic cardiovascular disease [19,20]. These successful applications underscore the versatility and efficacy of siRNA technology in addressing diverse medical conditions. The exquisite specificity of siRNAs enables targeting of virtually any gene, making them invaluable tools in both therapeutic applications and genomics research. This broad applicability has sparked intense interest in developing siRNA-based interventions for a multitude of genetic and acquired disorders. Recent research efforts have focused on improving siRNA delivery systems to enhance their therapeutic efficacy and broaden their application. For example, N-acetylgalactosamine (GalNAc)-conjugated siRNAs have shown promising results in facilitating efficient delivery to the liver, opening new avenues for treating liver-related diseases [21]. Additionally, advancements in lipid nanoparticle (LNP) technology are paving the way for systemic administration of siRNAs and their delivery to various tissues throughout the body. Innovative approaches combining CRISPR–Cas9 systems with siRNA technology are also being developed. These hybrid strategies hold the potential to simultaneously perform genome editing and gene expression suppression, enabling more sophisticated and nuanced genetic manipulations than previously possible. Despite their immense potential, siRNA technologies face several challenges that researchers are actively working to address. These include minimizing off-target effects, reducing immunogenicity (the propensity of RNA antigens to induce antibody production and cellular immune responses), and developing efficient delivery systems for targeting specific tissues. To overcome these hurdles, ongoing research focuses on improving chemical modification techniques, developing novel delivery systems, and optimizing siRNA design using AI algorithms. As the field of siRNA therapeutics continues to evolve, it promises to revolutionize the treatment of a wide range of diseases by offering highly specific and potent gene silencing capabilities. The continued refinement of this technology is expected to yield increasingly effective and safer therapeutic options, potentially transforming the landscape of molecular medicine in the coming years.

### 2.3. MicroRNAs (miRNAs)

MicroRNAs (miRNAs) occupy a pivotal position in the realm of RNA therapeutics [22]. These small non-coding RNA molecules, typically 21–25 nucleotides in length, are not translated into proteins but play crucial roles in gene regulation. The biogenesis of miRNAs involves a multistep process, beginning with the transcription of primary miRNAs (pri-miRNAs) that can span hundreds to thousands of bases. These pri-miRNAs undergo initial processing in the nucleus by the enzyme Drosha, resulting in precursor miRNAs (pre-miRNAs) with characteristic stem–loop structures. Following export to the cytoplasm, pre-miRNAs are further processed by the enzyme Dicer to yield mature miRNA molecules. Unlike siRNAs that typically target a single gene, miRNAs possess the remarkable ability to modulate entire gene networks. While miRNAs share the basic principle of binding to complementary sequences in target mRNAs with siRNAs, they primarily interact with the 3′-untranslated regions (3′-UTRs) of multiple target mRNAs. This unique characteristic enables miRNAs to exert broad regulatory effects on gene expression, influencing various cellular processes simultaneously. The significance of miRNA research was underscored by the awarding of the 2024 Nobel Prize in Physiology or Medicine to Victor Ambros and Gary Ruvkun for their groundbreaking discovery of miRNAs. Their initial work, which focused on developmental timing in nematodes, was initially considered a niche phenomenon. However, subsequent research revealed that miRNAs are evolutionarily conserved and play critical roles across numerous species, including humans. In the context of RNA therapeutics, miRNAs represent particularly attractive targets due to their involvement in diverse biological processes and their dysregulation in various diseases. Numerous studies have reported alterations in miRNA expression patterns associated with pathological conditions such as cancer, autoimmune disorders, and cardiovascular diseases [23]. Two primary approaches have emerged in miRNA-based therapeutic strategies. The first is miRNA replacement therapy, which aims to restore the function of miRNAs that are downregulated or lost in diseased tissues by introducing synthetic miRNA mimics. The second approach is miRNA inhibition therapy, which utilizes RNA molecules known as “anti-miRs” or “antagomirs” to bind to and inhibit the function of overexpressed miRNAs associated with disease progression [24]. The ability of miRNAs to target multiple genes simultaneously presents both opportunities and challenges in therapeutic development. On one hand, this multitargeting capability allows a single miRNA-based drug to potentially modulate several cellular processes concurrently, which can be particularly beneficial in treating complex diseases. For instance, in cancer therapy, targeting a single miRNA could potentially control multiple aspects of tumor biology, including proliferation, angiogenesis, and metastasis. On the other hand, this pleiotropic effect raises concerns about potential off-target effects and unintended consequences, as the regulation of numerous genes simultaneously may lead to unwanted side effects. To address these challenges, researchers are focusing on developing advanced delivery systems and improving the specificity of miRNA targeting. For example, progress is being made in the development of nanoparticle and exosome-based miRNA delivery systems, which promise more efficient and targeted delivery of miRNAs to specific tissues or cell types. Additionally, the application of AI algorithms is advancing miRNA target prediction and design optimization, potentially accelerating the development of more effective and safer miRNA-based therapeutics. As research in this field continues to evolve, miRNA-based therapies hold great promise for addressing a wide range of diseases by harnessing the natural regulatory mechanisms of these small but powerful RNA molecules. The ongoing efforts to refine miRNA delivery methods, improve target specificity, and enhance our understanding of miRNA biology are paving the way for innovative therapeutic approaches that could revolutionize the treatment of complex disorders in the coming years.

### 2.4. Messenger RNAs (mRNAs)

mRNA therapeutics have garnered significant attention in recent years, particularly following the success of COVID-19 mRNA vaccines. This innovative approach leverages the intrinsic role of mRNA as an intermediary between DNA and proteins, enabling the direct introduction of genetic information into cells to produce desired therapeutic proteins [25]. One of the most significant advantages of mRNA therapeutics is their ability to address “undruggable” targets that have traditionally been challenging for conventional pharmaceuticals and antibody-based therapies. The term “undruggable” refers to disease targets that are difficult to treat with traditional compounds due to their inability to bind or their unique structural characteristics. mRNA therapeutics hold immense potential, especially in treating diseases caused by deficient or dysfunctional proteins. For instance, in genetic disorders and metabolic syndromes, mRNA therapeutics can directly supplement missing proteins, offering a novel approach to treatment [26]. Unlike DNA-based gene therapies, mRNA therapeutics do not need to enter the cell nucleus. This characteristic substantially reduces the risk of integration into the genomic DNA. Furthermore, the transient nature of mRNA, which degrades after a certain period, allows for precise control over the duration of therapeutic effects. This feature is particularly crucial from a safety perspective, offering a significant advantage over other gene-based therapies. Advancements in mRNA design and delivery system technologies have dramatically improved the stability and translation efficiency of mRNA therapeutics. For example, developments in RNA chemical modification techniques have made it possible to reduce the immunogenicity of mRNA while enhancing its stability. Specific techniques employed include 5′ cap modification, optimization of poly(A) tails, and substitution with N1-methylpseudouridine. The development of lipid nanoparticle (LNP) delivery systems has enabled efficient delivery of mRNA to target cells. LNPs not only protect mRNA and facilitate cellular uptake but also aid in endosomal escape and release of mRNA into the cytoplasm, where it can be translated into therapeutic proteins [27]. Recent research has further expanded the scope of mRNA therapeutics. For instance, significant progress has been made in applying mRNA technology to cancer immunotherapy. Personalized cancer vaccines are being developed using mRNA encoding tumor-specific antigens or neoantigens (novel antigens arising from genetic mutations specific to cancer cells), aiming to activate the patient’s immune system against cancer. Current focus areas in mRNA therapeutic development include optimizing mRNA design, improving delivery systems for targeted tissue delivery, and controlling potential immunogenicity [28]. For example, research is ongoing to enhance mRNA expression efficiency and specificity by tissue-specific promoters and enhancer sequences, optimization of mRNA secondary structures, and modification of untranslated regions (UTRs). Additionally, the development of new delivery systems, such as exosomes and virus-like particles (VLPs), is progressing, promising more efficient and tissue-specific mRNA delivery. Regarding immunogenicity control, various approaches are being investigated, including suppression of Toll-like receptor (TLR) signaling and co-delivery of immunomodulatory molecules. These techniques are expected to further improve the safety and efficacy of mRNA therapeutics. The versatility of mRNA therapeutics opens possibilities for treating a wide range of diseases, from rare genetic disorders to more common conditions like cardiovascular diseases and metabolic disorders. As research in this field continues to advance, we can anticipate the development of increasingly sophisticated and targeted mRNA-based therapies, potentially revolutionizing the treatment landscape for numerous medical conditions.

### 2.5. Aptamers

**Figure 2:**
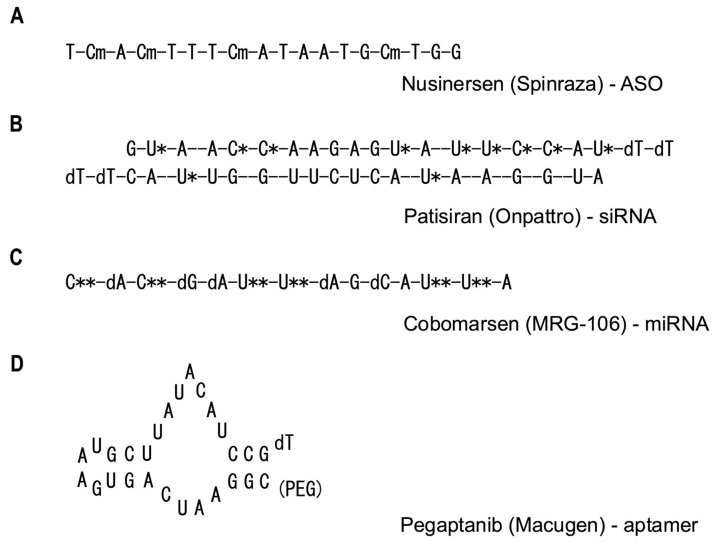
Structural representations of representative API molecules for ASO, siRNA, miRNA, and aptamer-based therapeutics. (**A**) Nusinersen, (**B**) patisiran, (**C**) cobomarsen, and (**D**) pegaptanib. In the figure, lowercase “m” denotes methylcytosine, “*” indicates 2′-*O*-methyl modifications, and “**” represents locked nucleic acid (LNA) modifications. These structural modifications enhance the stability and efficacy of the respective RNA therapeutics.

Aptamers are remarkable molecules that, despite being short single-stranded oligonucleotides, possess the ability to form complex three-dimensional structures. This unique characteristic enables aptamers to bind to a wide range of targets with high affinity and specificity [29]. In the field of RNA therapeutics, aptamers have gained a distinctive advantage by combining the specificity of antibodies with the benefits of synthetic oligonucleotides [30]. The versatile functionality of aptamers stems from their structural flexibility. These molecules can serve as antagonists (inhibitors), agonists (activators), or targeted ligands (signal molecules that bind to receptors and induce biological responses), making them valuable in various therapeutic modalities [31]. For instance, in cancer treatment, aptamers targeting specific cell surface proteins have been developed and are gaining attention as potential specific drug delivery systems to tumor cells. The structural adaptability of aptamers allows them to recognize and bind to a diverse array of targets, including small molecules, proteins, and even whole cells. This versatility is achieved through a process called systematic evolution of ligands by exponential enrichment (SELEX), which involves iterative rounds of selection and amplification to identify aptamers with the desired binding properties. The SELEX process can be fine-tuned to generate aptamers with specific characteristics, such as enhanced stability in biological fluids or improved tissue penetration. Innovative approaches combining aptamers with other RNA therapeutic technologies are advancing rapidly. Of particular interest is the development of aptamer–siRNA chimeric molecules. This approach aims to leverage the targeting capability of aptamers with the gene silencing power of siRNAs to achieve efficient delivery of siRNAs to target cells. By doing so, this technology holds promise for enabling more precise control of gene expression in the treatment of diseases such as cancer and genetic disorders. Improving tissue-specific delivery is another crucial focus of aptamer research. Various approaches are being explored, including combinations with nanoparticle technology and the addition of ligands targeting cell-specific receptors. These strategies aim to maximize the therapeutic effects of aptamers while minimizing potential side effects. For example, aptamers conjugated to nanoparticles can enhance the stability and circulation time of the therapeutic molecule, while cell-specific targeting can reduce off-target effects and improve efficacy. The development of diagnostic technologies utilizing aptamers is also progressing. Aptamer-based biosensors are gaining attention, particularly in the early detection of cancer and rapid detection of infectious diseases. These sensors enable highly sensitive and specific detection of target molecules, contributing to the realization of personalized medicine. The ability of aptamers to bind to a wide range of targets with high specificity makes them ideal candidates for developing point-of-care diagnostic tools that can rapidly and accurately detect biomarkers associated with various diseases. Furthermore, aptamers are being explored for their potential in theranostics, a field that combines therapeutic and diagnostic capabilities. In this context, aptamers can be designed to both detect disease markers and deliver therapeutic agents, offering a powerful tool for personalized medicine approaches. This dual functionality could lead to more efficient and targeted treatment strategies, potentially improving patient outcomes while reducing side effects [32]. As research in aptamer technology continues to advance, we can expect to see further innovations in their design, synthesis, and application. The integration of computational approaches, including AI, is likely to accelerate the development of aptamers with enhanced properties and functionalities. These advancements may lead to the creation of aptamers with improved stability, binding affinity, and specificity, further expanding their potential in both therapeutic and diagnostic applications (Table 1 and Figure 2).

## 3. Delivery Systems for RNA Drugs

### 3.1. Lipid Nanoparticles (LNPs)

Lipid nanoparticles (LNPs) have revolutionized RNA delivery systems, playing a pivotal role in the development of mRNA-based COVID-19 vaccines. These nanostructures, typically ranging from 50 to 200 nm in size, are composed of meticulously designed lipid mixtures that encapsulate and protect RNA molecules. The core of LNPs contains cationic lipids that electrostatically interact with negatively charged RNA, while helper lipids and polyethylene glycol (PEG)-conjugated lipids contribute to stability and extend circulation time. One of the most remarkable features of LNPs is their exceptional ability to facilitate cellular uptake and endosomal escape, which are crucial factors for the efficacy of RNA therapeutics. The adaptability of LNPs allows for fine-tuning of lipid composition, particle size, and surface characteristics, enabling the optimization of delivery systems for specific tissues or cell types. This versatility has been instrumental in advancing the field of RNA therapeutics. The advantages of LNPs are manifold, including efficient RNA encapsulation, enhanced cellular uptake, improved stability, and proven success in clinical applications, particularly in mRNA vaccines. These benefits have positioned LNPs at the forefront of RNA delivery technology, opening new possibilities for treating a wide range of diseases. However, LNPs are not without challenges. Some of the primary hurdles include limited biodistribution beyond the liver, potential immunogenicity concerns, and difficulties in large-scale manufacturing and quality control. Additionally, issues related to stability during storage and transportation, potential toxicity associated with cationic lipids, and the complexity of optimizing formulations for different RNA molecules remain ongoing challenges in the field. Current research efforts are focused on addressing these challenges to further expand the efficacy and applicability of LNP-based RNA therapeutics. Studies are being conducted to improve biodistribution beyond the liver and reduce potential immunogenicity, with the aim of broadening the therapeutic scope of LNP-based RNA treatments [33]. Furthermore, active research is underway to develop novel lipid compositions and surface modification techniques, optimize manufacturing processes, and enhance long-term stability [34,35,36]. These advancements are expected to solidify the role of LNPs in the field of RNA therapeutics and vaccines. There is excitement surrounding the potential expansion into broader medical applications, such as cancer treatment and gene therapy for inherited disorders. Simultaneously, there is a growing emphasis on carefully evaluating the balance between safety and efficacy, as well as further optimizing LNP technology for personalized medicine approaches. As the field progresses, researchers are exploring innovative strategies to overcome the current limitations of LNPs. For instance, the development of tissue-specific targeting ligands and the incorporation of stimuli-responsive elements into LNP formulations are being investigated to enhance targeted delivery and controlled release of RNA cargo. Additionally, the integration of computational modeling and high-throughput screening techniques is accelerating the design and optimization of LNP formulations, potentially leading to more efficient and tailored delivery systems [37]. The continued advancement of LNP technology is likely to have far-reaching implications for the future of medicine, potentially enabling more effective treatments for a wide range of diseases and contributing to the realization of personalized medicine. As research in this field intensifies, it is crucial for scientists and clinicians to collaborate closely to translate these technological innovations into safe and effective therapeutic interventions that can significantly improve patient outcomes.

### 3.2. Polymeric Nanoparticles (PNPs)

Polymeric nanoparticles (PNPs) have emerged as a versatile platform for RNA delivery, complementing LNPs in this crucial field [38]. These nanostructures, ranging from 10 to 1000 nm in size, are engineered from biodegradable and biocompatible polymers to encapsulate and protect RNA molecules. Both natural and synthetic polymers are utilized in PNP formulations, each offering unique advantages in terms of biocompatibility and physicochemical properties [39]. Cationic polymers have garnered particular attention in this context due to their ability to form stable complexes with RNA through electrostatic interactions. However, their application necessitates careful optimization to mitigate potential cytotoxicity. Recent advancements have led to the development of “stimuli-responsive polymeric nanoparticles”, which are designed to enhance stability in extracellular environments while facilitating intracellular RNA release [40]. The primary advantage of PNPs lies in their remarkable versatility. Researchers can tailor their size, charge, and surface properties to meet specific delivery requirements. Additionally, PNPs exhibit excellent stability, controlled release capabilities, and the potential for surface modification to enable targeted delivery. Their biodegradable nature also reduces the risk of long-term accumulation in the body. Despite these advantages, PNPs face several challenges. Concerns regarding the potential toxicity of cationic polymers persist, and achieving large-scale production remains difficult. The complexity of polymer–RNA interactions can complicate the prediction of in vivo behavior and optimization of formulations. Moreover, while stimuli-responsive designs show promise, precisely controlling release kinetics across diverse physiological environments remains challenging. To address these issues, ongoing research focuses on improving PNP design. This includes developing new biocompatible polymers and gaining a deeper understanding of polymer–RNA interactions. Researchers are also exploring the use of AI to streamline PNP design and optimization processes. Recent developments have seen increased interest in hybrid approaches that combine PNPs with other delivery systems. For instance, researchers are investigating hybrid nanoparticles that merge the characteristics of PNPs and LNPs, as well as systems that fuse PNPs with exosomes. These innovative approaches aim to leverage the strengths of each system while mitigating their respective limitations. Novel strategies for tissue-specific delivery are also being developed. These include attaching ligands to PNP surfaces to target receptors expressed on specific tissues or cells and developing advanced stimuli-responsive systems that release their payload in response to external stimuli such as light or magnetic fields. These advancements position PNPs as crucial players in the development of RNA therapeutics. Their potential applications span a wide range of medical fields, including cancer treatment, genetic disorder therapies, and vaccine development for emerging infectious diseases. As research progresses, PNP technology is expected to evolve further, establishing itself as an increasingly safe and effective RNA delivery system [41]. The future of PNPs in RNA delivery looks promising, with ongoing research addressing current limitations and exploring new frontiers. As our understanding of polymer–RNA interactions deepens and manufacturing processes improve, we can anticipate the emergence of more sophisticated and efficient PNP-based delivery systems. This progress will likely accelerate the translation of RNA therapeutics from laboratory concepts to clinical realities, potentially revolutionizing treatment approaches for a myriad of diseases.

### 3.3. Conjugation Strategies

Innovative conjugation strategies have emerged as a focal point in the field of RNA therapeutics, aiming to dramatically enhance the stability, delivery efficiency, and therapeutic efficacy of these promising molecules. These strategies involve the attachment of specific chemical structures or biomolecules to RNA molecules, with the goal of optimizing their pharmacokinetic properties and cellular uptake. One of the most prominent approaches in this arena is the N-acetylgalactosamine (GalNAc) conjugate. GalNAc has demonstrated high affinity for the asialoglycoprotein receptor, which is abundantly expressed on hepatocyte surfaces [42]. This natural binding affinity has been ingeniously exploited, proving to be exceptionally effective in targeting liver cells. The potential of GalNAc conjugates to revolutionize the treatment of liver-associated diseases has sparked considerable interest, with multiple clinical trials currently underway to explore their therapeutic applications. Antibody–oligonucleotide conjugates (AOCs) represent another promising strategy for enhancing the targeted delivery of RNA therapeutics. AOCs leverage the high specificity of antibodies to precisely target specific cell types or tissues. This approach not only improves the precision of therapy but also extends the circulation time of RNA molecules in the bloodstream [43]. Recent research has actively explored the application of AOCs in cancer treatment and rare disease therapies, positioning them as a crucial technology in the pursuit of personalized medicine. Lipid conjugation has also gained traction as an effective approach to enhance the membrane permeability and cellular uptake of RNA molecules. By binding RNA molecules to lipid moieties, researchers can facilitate the passage of RNA therapeutics across cell membranes, enabling more efficient intracellular delivery. This technology holds promise for improving delivery to tissues that have traditionally been challenging for RNA therapeutics to reach, such as the central nervous system and muscle tissues [44]. Efforts to further advance these conjugation strategies are ongoing. For instance, researchers are developing “smart delivery systems” by conjugating RNA molecules with environmentally responsive polymers, enabling the release of RNA molecules under specific pH conditions or in the presence of certain enzymes. Additionally, conjugation with cell-penetrating peptides is being investigated to further enhance cellular uptake. The fusion of nanotechnology with RNA conjugation strategies has also garnered significant attention. Researchers are developing novel delivery systems by conjugating RNA with nanomaterials such as gold nanoparticles and carbon nanotubes. These nanocomposites offer the potential for high stability and efficient cellular uptake, with the added possibility of incorporating stimulus-responsive drug release functionalities. These innovative conjugation strategies are expanding the potential applications of RNA therapeutics across a wide range of diseases, particularly in areas where traditional treatment approaches have fallen short, such as cancer, metabolic disorders, and neurodegenerative diseases. By enabling more effective targeting of RNA therapeutics to their intended sites of action while minimizing side effects, these approaches are opening new avenues for treatment. Looking ahead, key challenges include optimizing these conjugation strategies and evaluating their long-term safety profiles. Additionally, improving quality control in large-scale manufacturing and enhancing cost-efficiency remain important research priorities [45]. The concept of “personalized conjugation”, which involves customizing conjugation strategies based on individual patients’ genetic backgrounds and disease states, has also been proposed. This approach is driving research towards the realization of more precise personalized medicine. As the field of RNA therapeutics continues to evolve, these conjugation strategies are playing a pivotal role in overcoming traditional barriers to RNA delivery. The ongoing research and development in this area promise to unlock the full potential of RNA-based therapies, potentially revolutionizing treatment approaches for a wide array of diseases that have long eluded effective therapeutic interventions.

### 3.4. Viral Vectors

Viral vectors represent an exceptionally potent tool in the development of RNA therapeutics, ingeniously harnessing the innate ability of viruses to penetrate cells and deliver genetic material [46]. Among the most widely utilized viral vectors are adenoviruses, adeno-associated viruses (AAVs), and lentiviruses, each offering unique advantages in diverse applications within the field. AAV vectors have garnered significant attention due to their low immunogenicity and capacity to transduce a wide array of cell types. These vectors exhibit remarkable efficiency in delivering genetic material to various tissues, rendering them especially promising for the treatment of systemic diseases and central nervous system disorders. Recent advancements in AAV vector technology have focused on modifying surface proteins to achieve more specific tissue targeting, thereby enhancing their therapeutic potential and minimizing off-target effects. Lentiviral vectors, on the other hand, offer the advantage of enabling stable and long-term expression of RNA therapeutics. This characteristic makes them particularly valuable in scenarios where sustained therapeutic effects are crucial. Recent studies have demonstrated the immense potential of lentiviral vector-mediated genetic modification of hematopoietic stem cells in treating hereditary blood disorders, opening new avenues for gene therapy approaches. The development of hybrid systems that combine viral and non-viral components has emerged as an exciting area of research. These innovative systems aim to synergize the high transduction efficiency of viral vectors with the enhanced safety profile of non-viral carriers, thereby expanding the possibilities for RNA delivery. For instance, researchers are making significant progress in developing chimeric vectors that incorporate viral capsid proteins with synthetic polymers. This approach holds promise for reducing immunogenicity while simultaneously improving target specificity, addressing two critical challenges in viral vector-based therapies. The integration of viral vectors with genome editing technologies, particularly the CRISPR–Cas9 system, has opened new frontiers in gene therapy. By utilizing viral vectors to deliver components of the CRISPR–Cas9 system, researchers aim to achieve more efficient and sustained gene editing. The development of in vivo delivery systems for CRISPR–Cas9 using AAV vectors, for example, has the potential to revolutionize the treatment of genetic disorders by enabling precise genetic modifications directly within the patient’s body [47]. Despite their immense potential, viral vectors face several challenges that researchers are actively addressing. A primary focus is on enhancing the overall safety profile of these vectors. This includes the development of non-integrating vectors to minimize the risk of insertional mutagenesis, as well as the exploration of novel strategies to control immune responses. For instance, efforts are underway to modify viral capsid proteins to suppress the production of neutralizing antibodies, thereby enabling repeated administration of viral vector therapies without loss of efficacy. The application of AI in optimizing viral vector design represents another cutting-edge approach in the field. These computational tools are being employed to predict and enhance the efficiency and specificity of RNA delivery to target tissues and cells. By leveraging large datasets and complex algorithms, researchers aim to design viral vectors with improved tropism, reduced immunogenicity, and enhanced therapeutic payload capacity. As the field of viral vector-based RNA therapeutics continues to evolve, researchers are also exploring innovative production methods to address scalability and manufacturing challenges. The development of novel cell lines and bioreactor systems for large-scale vector production is crucial for translating promising pre-clinical results into clinically viable therapies. Furthermore, the combination of viral vectors with other delivery technologies, such as nanoparticles or exosomes, is being investigated to create hybrid delivery systems that capitalize on the strengths of multiple approaches. These hybrid systems may offer improved targeting, enhanced stability, and reduced immunogenicity compared to traditional viral vectors alone [48]. In conclusion, viral vectors remain at the forefront of RNA therapeutic development, offering unparalleled efficiency in genetic material delivery. As research progresses, the refinement of existing vectors and the development of novel hybrid systems promise to overcome current limitations, paving the way for more effective and safer RNA-based therapies across a wide spectrum of diseases. The advantages and disadvantages of each type of drug delivery system in this section are summarized in Table 2.

## 4. Clinical Applications and Approved RNA Drugs

### 4.1. Neurodegenerative Diseases

In the field of neurodegenerative diseases, RNA-based therapies have emerged as a promising approach for addressing conditions such as Alzheimer’s disease, Parkinson’s disease, Huntington’s disease, spinal muscular atrophy (SMA), and amyotrophic lateral sclerosis (ALS) [49]. The unique advantage of RNA therapeutics lies in their ability to target specific genes and molecular pathways involved in neurodegeneration. Approaches such as ASOs and RNAi have shown encouraging results in clinical trials for diseases like Huntington’s disease and SMA [50]. RNA-based therapies offer the potential to selectively reduce the expression of mutant proteins while maintaining the function of normal proteins, thus addressing the fundamental molecular mechanisms rather than merely treating symptoms. RNA therapeutics targeting tau, a microtubule-associated protein, are opening new possibilities for treating Alzheimer’s disease. For instance, an ASO-based RNA therapy called BIIB080 is currently in clinical trials, with reports indicating its ability to lower tau levels by more than 50%. In the case of Parkinson’s disease, RNA therapies aimed at suppressing the expression of the α-synuclein gene are being developed, with adeno-associated virus-mediated siRNA therapy showing promising results in pre-clinical stages. More precise RNA editing techniques are also being developed, such as targeted base editing using adenosine deaminase acting on RNA (ADAR) enzymes and the recruitment of endogenous ADAR using circular RNAs [51]. These novel technologies hold the potential to achieve higher specificity and efficiency in treating neurodegenerative diseases. However, significant challenges remain in the practical application of RNA therapeutics. The primary obstacles include crossing the blood–brain barrier and achieving widespread distribution within the central nervous system. To overcome these challenges, various strategies are being actively explored, including the use of chemically modified RNAs and novel delivery vectors. Innovative approaches such as exosome-mediated siRNA delivery and the development of oligonucleotides conjugated with cell-penetrating peptides are being attempted. The clinical application of RNA therapeutics is steadily progressing, with 27 RNA-based drugs currently in clinical trials. It is anticipated that further improvements in delivery technologies and the development of new RNA editing techniques will lead to revolutionary treatments for previously intractable neurodegenerative diseases. The advancements in RNA therapeutics are pushing the boundaries of neuroscience and medicine, holding the promise of significantly improving the quality of life for patients affected by these devastating conditions.

### 4.2. Genetic Disorders

RNA therapeutics are opening new avenues for the treatment of hereditary diseases, offering hope for numerous genetic disorders that have been challenging to address with conventional therapies. This innovative approach operates by modulating gene expression or replacing defective proteins, thereby illuminating potential solutions for previously intractable conditions. The core technologies underpinning RNA therapeutics encompass a diverse array of methodologies, including ASOs designed to correct errors in mRNA processing, siRNAs aimed at suppressing gene expression, and mRNA therapies intended to introduce functional proteins [52]. These methodologies have demonstrated promising outcomes in treating a wide spectrum of hereditary diseases. A notable example is the development and clinical application of siRNA-based drugs such as patisiran (Onpattro) and vutrisiran (Amvuttra) for the treatment of hereditary transthyretin-mediated amyloidosis [53]. These therapeutic agents have been shown to effectively slow disease progression by inhibiting the production of abnormal proteins, thereby offering tangible benefits to patients afflicted with this condition. The field of metabolic disorders has also witnessed significant advancements in RNA therapeutic applications. Research is progressing rapidly in the development of mRNA therapies for rare metabolic diseases such as methylmalonic acidemia and acute intermittent porphyria [54]. In these disorders, the introduction of mRNA encoding the deficient enzymes into the body holds promise for symptom amelioration, potentially transforming the treatment landscape for affected individuals. Furthermore, the application of RNA therapeutics in personalized medicine is gaining momentum. The ability to develop customized RNA drugs tailored to individual patients’ genetic mutations is paving the way for more precise and effective treatments. This approach offers new therapeutic options to patients who may have experienced limited success with conventional treatment modalities, thereby expanding the horizons of personalized medicine in the realm of genetic disorders. However, it is important to acknowledge that RNA therapeutics in the context of hereditary diseases still face several challenges that require resolution. These include concerns regarding long-term safety profiles, the durability of therapeutic effects, and the economic considerations of treatment costs. As research and development efforts continue to advance, it is anticipated that these hurdles will be overcome, solidifying RNA therapeutics as an established treatment modality that brings hope to an increasing number of patients affected by genetic disorders. The ongoing evolution of RNA therapeutics represents a paradigm shift in the approach to treating hereditary diseases. By directly addressing the underlying genetic causes of these conditions, RNA-based therapies offer the potential for more targeted and effective interventions. As our understanding of genetic mechanisms deepens and delivery technologies improve, the scope and efficacy of RNA therapeutics are likely to expand, potentially revolutionizing the treatment landscape for a wide array of genetic disorders.

### 4.3. Cancer Treatment

In the field of oncology, RNA therapeutics are being harnessed to target the intricate molecular mechanisms underlying tumor initiation and progression. A particularly promising avenue of research is the development of mRNA-based “cancer vaccines” designed to encode tumor-specific antigens. These innovative vaccines have shown encouraging results in stimulating the patient’s immune system to recognize and attack cancer cells, potentially revolutionizing cancer immunotherapy [55]. ASOs and RNAi technologies have emerged as powerful tools in cancer treatment. These approaches offer the potential to suppress specific oncogenes or restore the function of tumor suppressor genes, thereby addressing the genetic underpinnings of cancer development. The versatility of these RNA-based therapies allows for precise targeting of cancer-related genes, potentially minimizing off-target effects and improving therapeutic efficacy. mRNA therapeutics also hold significant promise in cancer treatment, offering the potential to directly deliver therapeutic proteins to cancer cells. This approach can be utilized for various purposes, such as replacing defective tumor suppressor proteins or introducing proteins that enhance the immune response against tumors [56]. The ability to produce these therapeutic proteins directly within the target cells could overcome some of the limitations associated with traditional protein-based therapies, such as poor stability or limited cellular uptake. Recent research has focused on combining RNA therapeutics with existing cancer treatments to overcome drug resistance and enhance therapeutic outcomes. This synergistic approach aims to leverage the strengths of multiple treatment modalities to achieve more comprehensive and durable responses in cancer patients. A notable example of this strategy was reported in a 2023 study, which demonstrated that combining an mRNA-based personalized cancer vaccine with immune checkpoint inhibitors significantly prolonged recurrence-free survival in patients with advanced melanoma [57]. This groundbreaking research underscores the potential of RNA therapeutics to complement and enhance the efficacy of established cancer treatments. By tailoring the mRNA vaccine to each patient’s unique tumor profile and combining it with immunotherapy, researchers were able to achieve a more potent and targeted antitumor immune response. This personalized approach represents a significant step forward in the field of precision oncology, offering hope for improved outcomes in patients with difficult-to-treat cancers. As research in RNA therapeutics continues to advance, we can anticipate further innovations in cancer treatment strategies. The flexibility and programmability of RNA-based therapies make them ideal candidates for developing highly specific and adaptable cancer treatments. Future directions may include the development of multitarget RNA therapies that simultaneously address multiple aspects of tumor biology or the creation of “smart” RNA therapeutics that can respond dynamically to the tumor microenvironment.

### 4.4. Infectious Diseases

In the realm of infectious diseases, RNA therapeutics have made remarkable strides, with the development and deployment of mRNA vaccines against COVID-19 serving as a particularly notable success story that has brought widespread recognition to the potential of this approach. The rapid development and global distribution of these vaccines have not only played a crucial role in mitigating the pandemic but have also demonstrated the versatility and efficacy of RNA-based interventions in addressing urgent public health challenges. Beyond vaccines, RNAi technology has emerged as a powerful tool in combating various viral infections. siRNAs designed to target specific viral genes and inhibit viral replication have garnered significant attention from researchers and clinicians alike [58]. These siRNAs offer the potential for highly targeted antiviral therapies that can be rapidly adapted to address emerging viral threats. Additionally, other RNA-based therapeutic approaches, such as ASOs and RNA aptamers, are being explored as potential solutions for antibiotic-resistant bacteria and viral infections, expanding the arsenal of tools available to combat infectious diseases [59]. A particularly noteworthy advantage of RNA therapeutics in the context of infectious diseases is the potential for rapid design and manufacturing. This agility in development and production allows for a nimbler response to emerging infectious threats, potentially reducing the time between the identification of a new pathogen and the availability of targeted interventions. Recent research efforts have focused on enhancing the target specificity and stability of RNA therapeutics, as well as improving their cellular delivery efficiency. These advancements are expected to facilitate the development of more effective treatments for a broader range of infectious diseases, potentially revolutionizing our approach to managing both endemic and epidemic pathogens. Furthermore, the integration of AI technologies into the field of RNA therapeutics has led to significant improvements in RNA sequence optimization and target selection. These computational approaches contribute to more accurate predictions of therapeutic efficacy and help minimize potential side effects, thereby enhancing the overall safety and effectiveness of RNA-based treatments. By leveraging these advanced technologies, researchers can more rapidly identify promising RNA therapeutic candidates and optimize their design for maximum efficacy against specific pathogens. The convergence of RNA therapeutics with other cutting-edge technologies is opening new avenues for personalized medicine in infectious disease treatment. For instance, the ability to rapidly sequence pathogens and design tailored RNA therapies could lead to more effective and targeted treatments for individual patients, potentially improving outcomes and reducing the risk of drug resistance. This personalized approach could be particularly valuable in managing complex or chronic infections that have proven resistant to conventional therapies. As RNA therapeutics continue to evolve, they are poised to play an increasingly important role in shaping the future of infectious disease management. The flexibility and precision of RNA-based approaches offer the potential to address long-standing challenges in infectious disease treatment, such as the emergence of drug-resistant pathogens and the need for rapid responses to novel infectious threats. By providing a new paradigm for infectious disease control, RNA therapeutics are expected to become an integral component of modern medical practice, offering hope for more effective, targeted, and adaptable interventions against a wide range of pathogens.

### 4.5. Cardiovascular Diseases

RNA therapeutics are opening new avenues for treating cardiovascular diseases by targeting the underlying molecular mechanisms [60]. This innovative approach offers promising solutions for patients who have not responded adequately to conventional pharmacological or surgical interventions. The field of RNA-based treatments in cardiovascular medicine encompasses a diverse range of strategies, each with unique potential to address specific aspects of heart and vascular health. siRNA drugs have demonstrated efficacy in managing hypertension and lowering LDL cholesterol levels, with several candidates progressing through clinical trials. These siRNA therapies work by selectively silencing genes involved in blood pressure regulation or cholesterol metabolism, offering a more targeted approach compared to traditional medications. For instance, inclisiran (Leqvio), an siRNA drug targeting PCSK9, has shown remarkable success in sustainably reducing LDL cholesterol levels with infrequent dosing, potentially improving patient adherence and outcomes in the long-term management of hypercholesterolemia [61]. ASOs are being investigated for their potential to target genes involved in lipid metabolism, providing novel approaches for treating familial hypercholesterolemia and other dyslipidemias. ASOs offer the advantage of high specificity and the ability to modulate gene expression at the RNA level, potentially addressing genetic factors underlying cardiovascular risk that have been challenging to target with conventional therapies. For example, ASOs targeting apolipoprotein B or lipoprotein have shown promise in clinical trials for reducing cardiovascular risk in patients with refractory hyperlipidemia. mRNA therapy is also gaining attention in cardiovascular medicine, with clinical trials underway for treatments such as vascular endothelial growth factor (VEGF) mRNA therapy aimed at promoting myocardial revascularization following myocardial infarction [62]. This approach leverages the body’s own cellular machinery to produce therapeutic proteins directly in the affected tissues, potentially offering more localized and sustained effects compared to traditional protein-based therapies. The ability to induce temporary and controlled expression of therapeutic proteins through mRNA delivery holds promise for addressing complex cardiovascular pathologies that require precise temporal and spatial regulation of protein expression. miRNA therapeutics are being explored for applications in treating atherosclerosis and coronary artery disease, among other cardiovascular conditions [63]. miRNAs play crucial roles in regulating gene expression networks involved in cardiovascular health and disease. By modulating these regulatory RNAs, researchers aim to influence multiple pathways simultaneously, potentially offering more comprehensive therapeutic effects. For example, miRNA therapies targeting inflammation, fibrosis, or angiogenesis pathways are being investigated for their potential to address the multifaceted nature of cardiovascular diseases. As research in RNA therapeutics advances, the development of personalized RNA treatments tailored to individual patients’ genetic backgrounds and disease characteristics is anticipated. This personalized approach holds the potential to revolutionize cardiovascular disease prevention and treatment by offering more precise and effective interventions. For instance, RNA-based therapies could be designed to address specific genetic variants associated with increased cardiovascular risk, providing targeted solutions for patients with inherited cardiovascular conditions. The integration of RNA therapeutics into cardiovascular medicine represents a paradigm shift in how we approach heart and vascular diseases. By directly addressing the molecular underpinnings of these conditions, RNA-based treatments offer the potential for more effective and durable therapeutic outcomes. As our understanding of cardiovascular genetics and molecular biology continues to grow, coupled with advancements in RNA delivery technologies, we can expect to see an expansion of RNA therapeutic options for cardiovascular patients in the coming years (Table 3).

## 5. Challenges in RNA Drug Development

### 5.1. Stability and Degradation

RNA stability presents a significant challenge in the development of RNA-based therapeutics. Natural RNA molecules are highly susceptible to degradation by ribonucleases present in biological fluids and tissues, resulting in short half-lives in vivo. This inherent instability has necessitated the development of various chemical modifications to enhance RNA stability and improve its therapeutic potential [64]. These modifications include the introduction of 2′-*O*-methyl, 2′-fluoro, and 2′-*O*-methoxyethyl groups, as well as the incorporation of locked nucleic acids (LNAs) and phosphorothioate linkages, as previously discussed [65]. In addition to chemical modifications, other strategies have been developed to improve RNA stability and control degradation. Nanoparticles, such as LNPs, can protect RNA molecules from degradation while simultaneously enhancing cellular uptake. These nanocarriers provide a protective environment for the RNA, shielding it from nucleases and other degradative factors in the biological milieu. Conjugation strategies, involving the attachment of RNA to molecules like cholesterol or N-acetylgalactosamine, can also enhance RNA stability and promote targeted delivery to specific tissues or cell types. Furthermore, the design of RNA molecules with specific secondary and tertiary structures can improve their resistance to degradation by creating more compact and stable conformations that are less accessible to nucleases. Understanding and controlling RNA stability and degradation are crucial for pharmaceutical development. Enhancing RNA stability can extend the half-life of RNA therapeutics, potentially reducing dosing frequency and improving patient compliance. This is particularly important for chronic conditions that require long-term treatment, where less frequent administration can significantly improve quality of life for patients. Moreover, controlled degradation can be harnessed as a therapeutic strategy, especially when targeting disease-related RNAs. By designing RNA molecules that are selectively degraded in specific cellular environments or in response to stimuli, it is possible to create more precise and targeted therapeutic approaches. The study of RNA stability and degradation is laying the groundwork for the realization of personalized medicine and precision therapeutics. As our understanding of RNA biology and chemistry continues to advance, we can anticipate the development of more sophisticated and tailored RNA-based treatments. For instance, RNA therapeutics could be designed with specific stability profiles that match the pharmacokinetic requirements of individual patients or disease states. This level of customization could lead to more effective and safer RNA-based therapies, potentially offering new treatment options for a wide range of diseases, including those that have been challenging to address with conventional therapeutic approaches. Looking ahead, the continued advancement of technologies for enhancing RNA stability and controlling degradation is expected to drive the development of increasingly effective and safe RNA-based therapeutics. These innovations may include novel chemical modifications, more advanced delivery systems, and sophisticated RNA designs that respond dynamically to biological cues. As these technologies mature, we can anticipate a new era of RNA therapeutics that offer unprecedented precision in targeting disease mechanisms, potentially revolutionizing treatment paradigms across multiple medical fields.

### 5.2. Off-Target Effects

Off-target effects represent a significant challenge in RNA therapeutics, particularly in approaches based on RNAi technology. These undesirable effects occur when therapeutic RNA molecules interact with and modulate the expression of genes other than their intended targets, potentially leading to unforeseen consequences and safety concerns. In RNAi-based therapies, off-target effects primarily arise from partial complementarity between siRNAs or miRNAs and unintended mRNA targets [66]. The seed region, comprising nucleotides 2–7 or 2–8 at the 5′ end of the guide strand, plays a crucial role in target recognition. Even short, limited complementarity within this region can potentially induce unintended gene silencing. To mitigate these off-target effects, researchers have developed various strategies. Chemical modifications of RNA molecules, such as 2′-*O*-methyl modifications, can enhance specificity [67]. Pooling multiple siRNAs targeting the same gene can maintain efficacy while reducing individual off-target effects. Furthermore, careful RNA sequence design, particularly through comprehensive bioinformatics analysis to minimize potential off-target binding sites, has become essential in developing more specific RNA therapeutics. Recent advancements in RNA editing technology, such as the CRISPR–Cas13 system, have shown promise in achieving higher specificity and efficiency [68]. This approach allows for the design of highly specific guide RNAs targeting the RNA of interest, significantly reducing off-target effects. Additionally, the optimization of sequence design through AI algorithms is progressing, contributing to the prediction and avoidance of off-target effects. While off-target effects remain a critical challenge in the clinical application of RNA therapeutics, ongoing technological innovations and research advancements are continuously evolving methods to minimize their impact. The field is moving towards more sophisticated approaches, such as personalized treatment designs that consider individual patient genomic information and the development of more precise delivery systems utilizing nanotechnology. These multifaceted approaches are expected to further enhance the safety and efficacy of RNA therapeutics. The complexity of off-target effects underscores the importance of comprehensive pre-clinical studies and rigorous safety assessments in the development of RNA-based therapies. Researchers are increasingly employing advanced transcriptomics and proteomics techniques to evaluate the global impact of RNA therapeutics on gene expression and cellular function. These holistic approaches provide a more complete picture of potential off-target effects and help in refining therapeutic strategies. Moreover, the development of novel RNA chemistries and structures is an active area of research aimed at enhancing target specificity. For instance, the design of aptamers or structured RNAs that can only bind to their intended targets with high affinity and specificity offers a promising avenue for reducing off-target effects. These highly specific RNA molecules could potentially be used as therapeutic agents themselves or as delivery vehicles for other RNA-based drugs. As our understanding of RNA biology and cellular mechanisms deepens, new strategies for controlling and exploiting off-target effects may emerge. For example, some researchers are exploring the potential of deliberately inducing specific off-target effects as part of a therapeutic strategy, particularly in complex diseases where modulating multiple pathways simultaneously might be beneficial. In conclusion, while off-target effects pose a significant challenge in RNA therapeutics, the field is rapidly evolving to address this issue. Through a combination of innovative design strategies, advanced delivery systems, and cutting-edge analytical techniques, researchers are paving the way for safer and more effective RNA-based therapies. As these technologies continue to mature, we can anticipate the development of RNA therapeutics with increasingly precise targeting capabilities, opening new possibilities for treating a wide range of diseases with greater efficacy and reduced side effects.

### 5.3. Immunogenicity

Immunogenicity in RNA therapeutics is a critical consideration that significantly impacts both safety and efficacy [69]. RNA molecules possess an inherent immunogenicity, capable of activating pattern recognition receptors of the innate immune system. While this immunostimulatory property may be advantageous for RNA vaccines, it can potentially lead to reduced efficacy and safety concerns in other RNA therapeutics aimed at protein replacement or gene regulation. To address immunogenicity issues, strategies have been developed to modulate immune responses to RNA molecules. One of the most effective approaches involves the introduction of modified nucleosides such as pseudouridine, 5-methylcytidine, and N6-methyladenosine [70]. These modifications can substantially reduce the activation of pattern recognition receptors, thereby suppressing immune responses and enhancing the translation efficiency of therapeutic mRNAs. The incorporation of these modified nucleosides has been instrumental in improving the tolerability and effectiveness of RNA-based therapies. The delivery systems employed for RNA therapeutics also play a crucial role in their immunogenicity profile. LNPs, commonly used for encapsulation and delivery of RNA molecules, have been noted to potentially elicit immune responses themselves. Optimizing the composition and physicochemical properties of nanoparticles can help minimize immunogenicity while maintaining efficient RNA delivery. This delicate balance between effective delivery and immune evasion represents an ongoing area of research and development in the field. Sequence optimization and structural design of RNA molecules have also been recognized as important factors in controlling immunogenicity. Avoiding specific sequence motifs or secondary structures known to trigger immune responses, or incorporating immunosuppressive sequences, can further modulate the immune response. Additionally, personalized approaches that customize RNA therapeutics based on individual patients’ genetic backgrounds and immune status represent promising future strategies for mitigating immunogenicity issues. As our understanding of RNA therapeutic immunogenicity deepens, it paves the way for the development of safer and more effective treatments. However, fully controlling the complex interactions with the immune system remains a challenge, necessitating ongoing research and clinical trials to ensure long-term safety and efficacy. The continued exploration of novel modification techniques, delivery systems, and methods for more precise control of immune interactions will undoubtedly contribute to the further advancement and expanded clinical application of RNA therapeutics. The multifaceted nature of RNA immunogenicity requires a comprehensive approach to its management. Researchers are increasingly employing advanced immunological assays and in silico prediction tools to assess and mitigate potential immunogenicity risks during the early stages of RNA therapeutic development. These tools allow for the identification of immunogenic epitopes and the design of RNA sequences with reduced potential for immune activation. Moreover, the interplay between RNA therapeutics and the adaptive immune system is an area of growing interest. While much focus has been placed on innate immune responses, the potential for RNA therapies to induce adaptive immune responses, including the production of antidrug antibodies, is also being closely examined. Understanding and mitigating these long-term immunological effects will be crucial for the success of chronic or repeated RNA therapeutic administrations. The development of novel RNA chemistries and structures continues to be a frontier in addressing immunogenicity. For instance, circular RNAs and self-amplifying RNAs are being explored for their potential to offer prolonged expression with reduced immunogenicity [71]. These innovative RNA formats may provide new avenues for developing therapeutics with improved safety profiles and enhanced efficacy. As the field of RNA therapeutics continues to evolve, the management of immunogenicity will likely become increasingly sophisticated and tailored. The integration of immunomodulatory strategies, such as co-administration of immune suppressants or the use of tolerogenic nanoparticles, may offer additional tools for controlling unwanted immune responses. Furthermore, the potential for exploiting the immunostimulatory properties of RNA in a controlled manner, particularly in the context of cancer immunotherapy or vaccine development, represents an exciting area of future research. In conclusion, while immunogenicity presents a significant challenge in the development of RNA therapeutics, it also offers opportunities for innovation and precision medicine. The ongoing advancements in our understanding of RNA–immune interactions, coupled with technological innovations in RNA design and delivery, promise to usher in a new era of RNA therapeutics with optimized safety and efficacy profiles. As research progresses, we can anticipate the emergence of more sophisticated, patient-tailored RNA therapies that harness the full potential of this versatile molecular platform while minimizing unwanted immune responses.

### 5.4. Delivery to Target Tissues

Effective delivery of RNA molecules to specific tissues and cell types is crucial for maximizing the therapeutic efficacy of RNA-based treatments while minimizing off-target effects and potential toxicity. To overcome the inherent instability of RNA in biological systems and facilitate targeted delivery, a diverse array of delivery systems has been developed and refined over the years. LNPs have emerged as a particularly promising delivery vehicle for RNA therapeutics. These nanoparticles encapsulate RNA molecules, shielding them from degradation and enabling efficient cellular uptake. The versatility of LNPs allows for the optimization of their composition to enhance tissue-specific targeting. By fine-tuning the lipid components, surface properties, and size of LNPs, researchers can modulate their biodistribution and cellular internalization profiles. For instance, incorporating specific lipids or attaching targeting ligands to the LNP surface can promote preferential uptake by certain cell types or tissues [72]. Conjugate-based approaches have also demonstrated significant potential for targeting specific organs or cell types. A notable example is the conjugation of GalNAc to siRNA molecules, which has proven highly effective for liver-specific delivery. This strategy exploits the high expression of asialoglycoprotein receptors on hepatocytes, enabling efficient and selective uptake of GalNAc-conjugated RNA therapeutics by liver cells. The success of this approach has led to the development of several clinically advanced RNA therapeutics targeting liver-expressed genes. The route of administration plays a crucial role in tissue-specific delivery as well. While intravenous injection for systemic delivery is common, local administration methods such as intramuscular, subcutaneous, or intratumoral injections can enhance delivery to specific tissues while reducing systemic exposure [73]. These localized delivery approaches can be particularly beneficial for targeting diseases with well-defined anatomical locations or for minimizing off-target effects in non-target tissues. Advancements in RNA chemistry have also contributed to improved delivery and targeting. Chemical modifications of RNA molecules, such as the incorporation of 2′-*O*-methyl or 2′-fluoro groups, not only enhance stability but can also influence tissue distribution and cellular uptake. These modifications can alter the pharmacokinetic properties of RNA therapeutics, potentially leading to improved target engagement and reduced off-target effects. The development of cell-type-specific promoters and regulatory elements has opened new possibilities for targeted RNA expression. By incorporating these elements into mRNA or self-amplifying RNA constructs, it is possible to restrict therapeutic RNA expression to specific cell types or tissues, even after systemic administration. This approach combines the benefits of broad distribution with highly specific activation in target cells. Exosomes and extracellular vesicles represent another frontier in RNA delivery. These naturally occurring nanoparticles can be engineered to carry therapeutic RNAs and possess intrinsic tissue-targeting properties. By harnessing the natural trafficking mechanisms of exosomes, researchers aim to develop more efficient and specific RNA delivery systems. As the field of RNA therapeutics continues to evolve, the integration of advanced technologies such as AI is expected to play an increasingly important role in optimizing delivery strategies [74]. These computational approaches can help predict the behavior of RNA therapeutics in vivo and guide the design of more effective delivery systems tailored to specific therapeutic applications. In conclusion, the development of efficient and targeted RNA delivery systems remains a critical area of research in the field of RNA therapeutics. The ongoing advancements in nanoparticle technology, conjugate chemistry, and our understanding of cellular uptake mechanisms are paving the way for more precise and effective RNA-based treatments. As these delivery technologies continue to improve, we can anticipate the emergence of RNA therapeutics with enhanced tissue specificity, improved efficacy, and reduced side effects, ultimately expanding the range of diseases that can be effectively treated with this innovative therapeutic modality (Table 4).

## 6. Emerging Technologies and Approaches

### 6.1. Chemical Modifications

The efficacy and safety of RNA-based therapeutics are heavily dependent on chemical modifications that enhance stability, reduce immunogenicity, and improve pharmacokinetic properties. One crucial chemical modification involves the incorporation of modified nucleosides, such as pseudouridine and N1-methyl-pseudouridine, which significantly diminish the immunostimulatory effects of RNA molecules [75]. These alterations enable the evasion of recognition by Toll-like receptors, resulting in decreased inflammatory responses and increased protein expression in vivo. Chemical modifications of the ribose moiety play a pivotal role in enhancing RNA stability and reducing susceptibility to nuclease degradation. Specific chemical modifications include 2′-*O*-methyl, 2′-fluoro, and 2′-*O*-methoxyethyl groups [76]. These modifications not only improve the pharmacokinetic properties of RNA therapeutics but also contribute to reduced immunogenicity. The intricate interplay between these modifications and RNA structure–function relationships is an area of ongoing research in the field of nucleic acid therapeutics. Phosphate modifications, such as phosphorothioate linkages, are employed to increase resistance to nuclease degradation and improve the pharmacokinetic profile of RNA drugs [77]. These chemical alterations can significantly extend the half-life of RNA molecules in biological fluids and tissues. The incorporation of phosphorothioate linkages has been shown to enhance the stability of various RNA-based therapeutics, including antisense oligonucleotides and siRNAs. In mRNA-based therapeutics, cap modifications are of paramount importance. Synthetic cap analogs, such as antireverse cap analogs, ensure proper orientation during in vitro transcription and enhance translation efficiency. Moreover, optimizing the length and composition of the poly(A) tail can substantially improve the performance of mRNA therapeutics. These modifications work synergistically to increase mRNA stability, translation efficiency, and overall therapeutic efficacy [78]. Furthermore, codon optimization of mRNA represents another strategy to enhance protein expression without altering the amino acid sequence of the encoded protein. This technique improves translation efficiency by adjusting codon usage to match that of the target tissue. The process involves selecting synonymous codons that are more frequently used in the host organism, thereby potentially increasing the rate of protein synthesis and overall yield. Codon optimization has been successfully applied in the development of various mRNA-based vaccines and therapeutics, demonstrating its potential to enhance the efficacy of these novel treatment modalities.

### 6.2. Novel Delivery Platforms

In the realm of RNA therapeutics development, the establishment of effective delivery systems remains a paramount challenge. To surmount this obstacle, a diverse array of delivery platforms has been engineered, with extracellular vesicles, particularly exosomes, garnering significant attention. Exosomes possess an inherent ability to traverse biological barriers and exhibit low immunogenicity, making them highly promising candidates. Furthermore, researchers are exploring methods to engineer exosomes for the encapsulation of therapeutic RNA and targeted delivery to specific cell types. This approach may offer a more biocompatible alternative to synthetic nanoparticles, potentially revolutionizing the field of RNA drug delivery. “Biomimetic nanoparticles” represent an innovative fusion of synthetic nanoparticle advantages with the benefits of biological components, such as cell membranes. For instance, nanoparticles coated with erythrocyte membranes have demonstrated encouraging results in extending circulation time and reducing immunogenicity [79]. These hybrid structures leverage the natural properties of cellular components to enhance the performance of synthetic delivery systems, potentially bridging the gap between artificial and biological approaches to RNA delivery. Hydrogels have emerged as versatile platforms for localized and sustained delivery of RNA therapeutics [80]. These hydrophilic polymers can be engineered to encapsulate RNA molecules and release them in a controlled manner. Injectable hydrogels offer significant advantages for local delivery, as they can form in situ and provide sustained RNA release. The tunable properties of hydrogels allow for customization of release kinetics and degradation profiles, making them adaptable to various therapeutic applications and tissue environments [81]. Cell-based delivery systems represent a cutting-edge frontier in RNA therapeutics. This approach utilizes living cells, such as immune cells or stem cells, as vehicles for delivering RNA to specific tissues or tumors. These cells are engineered ex vivo to either produce and secrete exosomes loaded with RNA or directly deliver RNA upon reaching their target. The use of cell-based delivery systems capitalizes on the natural homing and tissue-penetrating abilities of certain cell types, potentially overcoming barriers that limit the efficacy of conventional delivery methods. The development of these advanced delivery platforms is driven by the need to address the limitations of traditional RNA delivery methods. Each approach offers unique advantages and challenges, and their successful implementation could significantly expand the therapeutic potential of RNA-based drugs. As research in this field progresses, it is likely that combination strategies, incorporating elements from multiple delivery platforms, will emerge to create more effective and tailored RNA delivery systems for a wide range of medical applications [82].

### 6.3. Combination Therapies

In the field of RNA therapeutics, combination therapy has emerged as a promising approach to overcome the limitations of monotherapy and achieve enhanced therapeutic efficacy. This strategy involves combining multiple RNA therapeutics or integrating RNA therapeutics with conventional drugs and treatment modalities [83]. The primary advantage of combination therapy lies in its ability to simultaneously target multiple pathways and mechanisms. This approach is particularly valuable in treating complex diseases, such as cancer, which often develop resistance to single-agent therapies. By combining different RNA molecules, such as siRNAs targeting distinct oncogenes or miRNAs regulating various cellular processes, researchers can now develop more comprehensive and effective treatment strategies [84]. In cancer treatment, the combination of RNA therapeutics with traditional chemotherapeutic agents has demonstrated synergistic effects, potentially enhancing the efficacy of chemotherapy while mitigating drug resistance [85]. For instance, the co-administration of siRNAs targeting multidrug resistance genes with chemotherapeutic drugs has shown promising results in overcoming chemoresistance in various cancers. Recent studies have further evolved this combinatorial approach, exploring new possibilities such as enhancing immunotherapy by combining immune checkpoint inhibitors with RNA therapeutics. This innovative strategy aims to modulate the immune response and improve the overall efficacy of cancer treatment. Advanced nanoparticle systems, such as LNPs and polymeric nanocarriers, can be engineered to encapsulate and simultaneously deliver multiple RNA molecules or combinations of RNA and small-molecule drugs [86]. These sophisticated delivery systems not only protect RNA from degradation but also enable targeted delivery to specific tissues or cell types, thereby enhancing the therapeutic potential of combination therapies. Recent research has focused on incorporating tumor-microenvironment-responsive features into these nanoparticle systems, allowing for more precise control over drug release and improved therapeutic outcomes. The prospects of combination therapy in RNA therapeutics are particularly exciting. Researchers are exploring the use of AI algorithms to predict optimal combinations and develop personalized treatment regimens based on individual patients’ genetic profiles. Additionally, efforts are underway to develop novel therapeutic strategies that combine RNA therapeutics with genome editing technologies such as CRISPR–Cas9. These innovative approaches are expected to further expand the possibilities of combination therapy in RNA therapeutics, accelerating the development of more effective and safer treatment options for a wide range of diseases. As the field of RNA therapeutics continues to evolve, combination therapy approaches are likely to play an increasingly important role in addressing complex diseases and overcoming therapeutic challenges. The integration of advanced delivery systems, personalized medicine approaches, and cutting-edge technologies promises to unlock new possibilities in RNA-based combination therapies, potentially revolutionizing the treatment landscape for numerous medical conditions.

### 6.4. Personalized RNA Medicines

**Figure 3:**
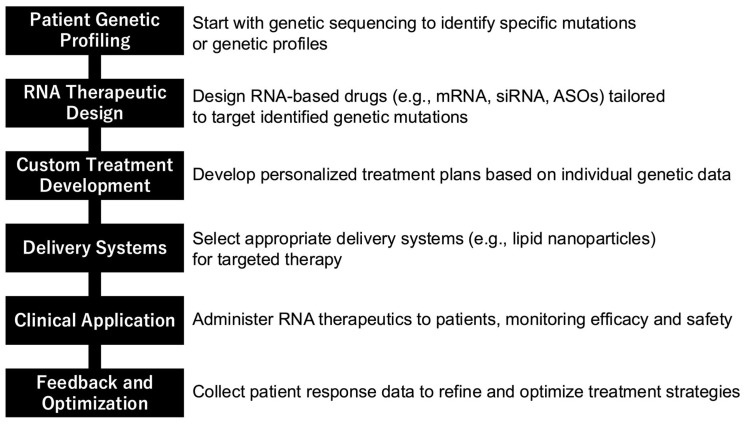
A detailed flowchart depicting the role of RNA therapeutics in personalized medicine. It outlines the process from genetic profiling to tailored treatment strategies, emphasizing the precision and adaptability of RNA-based approaches in addressing individual genetic variations and disease profiles.

Personalized RNA therapeutics represent a groundbreaking frontier in RNA-based treatments [87], holding the potential to revolutionize patient care through customized therapies based on individual genetic profiles (Figure 3). This approach harnesses the inherent flexibility of RNA molecules to address specific genetic mutations and abnormalities unique to each patient. The concept of personalized RNA therapeutics is particularly promising in cancer treatment, where mRNA-based cancer vaccines designed specifically for a patient’s oncogenic profile have been developed and are showing encouraging results in clinical trials [88]. The rapid adaptability of RNA therapeutics is a crucial advantage in personalized medicine. Unlike traditional pharmaceuticals or biopharmaceuticals, RNA therapeutics can be swiftly modified by simply altering the nucleotide sequence [89]. This flexibility enables the rapid development of treatments tailored to individual genetic variations or mutations, with the process potentially completed in a matter of weeks [90]. In the realm of rare genetic disorders, personalized RNA therapeutics are offering hope for conditions long considered untreatable. The success of milasen in 2019, a drug created for a single patient with a rare neurodegenerative disease, exemplifies the potential of this approach [91]. By sequencing the patient’s genome and identifying the specific mutation causing the disease, researchers were able to design and manufacture a personalized ASO targeting this mutation in less than a year. As research in this field progresses, we can envision a future where genetic information routinely influences treatment decisions, with RNA therapeutics playing a central role in delivering truly personalized medicine. The ongoing advancements in RNA therapeutics are poised to revolutionize the treatment of a wide range of diseases, offering new hope to patients and transforming the landscape of modern medicine. As of 2024, the latest developments in the field show a surge in clinical trials for personalized RNA therapeutics, with notable progress particularly in the areas of rare diseases and refractory cancers. The integration of AI has further streamlined the design of optimal RNA sequences for individual patients, significantly reducing the development time for these tailored therapies. This technological synergy is accelerating the translation of personalized RNA therapeutics from concept to clinical application. Moreover, advancements in delivery systems specifically designed for personalized RNA therapeutics have addressed many of the challenges associated with targeted delivery and cellular uptake. Novel nanoparticle formulations and engineered extracellular vesicles are enhancing the precision and efficacy of these personalized treatments, allowing for more accurate targeting of specific tissues or cell types. The regulatory landscape is also evolving to accommodate the unique challenges posed by personalized RNA therapeutics. Regulatory agencies are developing new frameworks to assess the safety and efficacy of these highly individualized treatments, balancing the need for rigorous evaluation with the urgency often associated with rare and life-threatening conditions. As we look to the future, the integration of personalized RNA therapeutics with other cutting-edge technologies, such as gene editing and regenerative medicine, promises to open new avenues for treating previously intractable diseases. This convergence of technologies is likely to usher in a new era of precision medicine, where treatments are not just personalized but are dynamically adapted to the evolving needs of each patient throughout their treatment journey.

## 7. Regulatory Considerations and Clinical Trials

**Figure 4:**
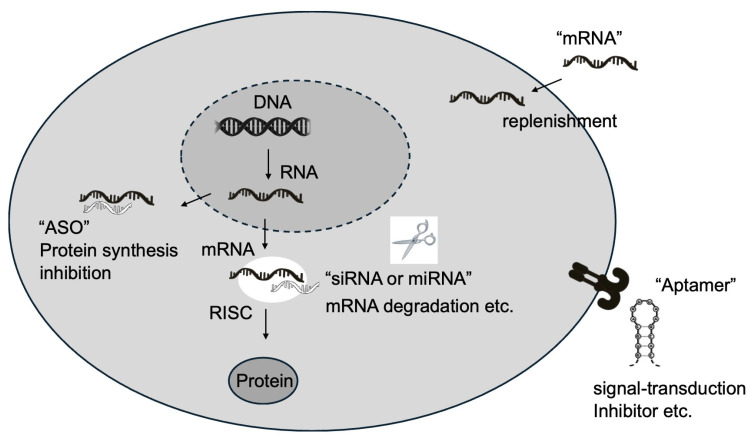
An overview of diverse RNA therapeutic applications across various diseases. This figure illustrates how different RNA modalities, including ASOs, siRNAs, miRNAs, mRNA vaccines, and aptamers, target specific medical conditions through unique mechanisms, highlighting their versatility in addressing a wide range of diseases.

The field of RNA therapeutics is rapidly evolving, with regulatory considerations and clinical trials playing pivotal roles in translating these innovative therapies from laboratory to patient care [92]. The unique nature of RNA therapeutics presents distinct challenges from a regulatory perspective, necessitating sophisticated approaches from both regulatory authorities and pharmaceutical developers. Regulatory agencies such as FDA and EMA are compelled to adapt their regulatory frameworks to accommodate the novel mechanisms and manufacturing processes of RNA therapeutics. These organizations are working diligently to establish guidelines that ensure the safety and efficacy of RNA therapeutics while fostering innovation in this promising field. One of the primary regulatory considerations for RNA therapeutics is their classification. Depending on their specific mechanism of action and intended use, RNA drugs may be categorized as gene therapy products, biological products, or chemical entities. This classification has significant regulatory implications, influencing the type of marketing authorization required and the specific controls that must be implemented throughout the drug development process. The rapid adaptability of RNA therapeutics presents new challenges. While the ability to quickly modify RNA sequences to target different diseases or variants offers the potential for accelerated development timelines, it also raises questions about how to assess the safety of rapidly developed products. Regulatory authorities must develop frameworks that can accommodate this agility while maintaining rigorous safety standards. Clinical trials for RNA therapeutics have unique aspects compared to those for conventional drugs and biopharmaceuticals. A key consideration is the potential for off-target effects, which can be challenging to predict and evaluate. Consequently, clinical trial designs for RNA therapeutics often incorporate extensive safety monitoring and long-term follow-up to detect unexpected effects. The success of mRNA vaccines during the COVID-19 pandemic has significantly influenced the regulation of RNA therapeutics. The emergency use authorizations granted to mRNA vaccines demonstrated the potential for expedited approval pathways for RNA therapeutics in situations of urgent need. This has sparked discussions about how similar approaches might be applied to other RNA therapeutics, particularly for rare diseases or conditions with high unmet medical needs. Manufacturing considerations also play a crucial role in the regulation of RNA therapeutics. The production of RNA drugs often involves novel processes, such as in vitro transcription, which require specialized quality controls. Regulatory authorities recognize the importance of collaborating with manufacturers to establish appropriate standards for the production and clinical testing of RNA therapeutics. As the field of RNA therapeutics continues to advance, there is an increasing need for international harmonization of regulatory approaches. Organizations such as the International Council for Harmonisation of Technical Requirements for Pharmaceuticals for Human Use (ICH) are working towards developing global standards for the development and regulation of RNA therapeutics. The regulatory landscape for RNA therapeutics is dynamic and evolving. Regulatory agencies are continuously refining their approaches to address the unique challenges posed by these innovative therapies [93]. This includes developing new guidelines for the evaluation of long-term safety, establishing appropriate biomarkers for efficacy assessment, and creating flexible regulatory pathways that can accommodate the rapid pace of innovation in this field. In conclusion, the regulation of RNA therapeutics represents a complex and evolving landscape that requires ongoing collaboration between regulatory authorities, pharmaceutical companies, and academic researchers. As our understanding of RNA therapeutics grows and new applications emerge, regulatory frameworks will continue to adapt to ensure that these promising therapies can be safely and effectively brought to patients while fostering continued innovation in this groundbreaking field of medicine (Figure 4).

## 8. Future Perspectives and Conclusions

RNA therapeutics are opening revolutionary frontiers in medicine and drug development, harboring the potential to transform the landscape of healthcare. As we look to the future, several critical perspectives emerge, highlighting the immense possibilities and challenges in this field. One of the most promising aspects of RNA therapeutics is their applicability to personalized medicine. The ability to rapidly design and manufacture RNA-based drugs tailored to an individual’s genetic profile offers unprecedented possibilities for treating complex diseases such as rare genetic disorders and cancer.

The case of milasen, created in less than a year for a patient with a rare neurodegenerative disease, exemplifies how RNA therapeutics can respond swiftly and precisely to unmet medical needs. The diversity of RNA as a therapeutic modality is also a key factor driving its future potential. From mRNA vaccines to ASOs, siRNAs, and aptamers, RNA therapeutics can target a wide range of diseases through various mechanisms. This flexibility, coupled with advancements in delivery technologies such as LNPs, is expanding the range of treatable diseases and the targeting of “undruggable” targets. The COVID-19 pandemic has served as a catalyst for RNA therapeutics, particularly in mRNA vaccines, demonstrating the rapid development and deployment capabilities of this technology. This success is likely to accelerate research into RNA-based approaches for other infectious diseases and non-communicable conditions. In the future, the integration of RNA therapeutics with other cutting-edge technologies is expected to further enhance their potential. For instance, combining RNA therapeutics with gene editing technologies could lead to more precise and effective gene therapies. Moreover, the application of AI in the design and optimization of RNA therapeutics is anticipated to streamline the development process and improve efficacy. However, several challenges remain in realizing the full potential of RNA therapeutics. These include improving delivery systems to optimize distribution to target tissues, mitigating potential off-target effects, and ensuring long-term safety. Regulatory frameworks will also need to evolve to accommodate the unique aspects of RNA-based drugs, particularly for personalized therapies. Looking ahead, we may see the emergence of RNA therapeutic platforms within healthcare institutions. Hospital-based RNA therapy programs could facilitate the rapid development and clinical application of personalized treatments, bringing cutting-edge therapies closer to patients. This suggests a new form of decentralized and personalized medicine. The future may also see advancements in the scalability and cost-effectiveness of RNA therapeutic production. As manufacturing processes become more efficient and streamlined, the accessibility of these treatments could significantly increase, potentially democratizing access to highly personalized medicine. Furthermore, the convergence of RNA therapeutics with other emerging fields, such as nanotechnology and tissue engineering, could open new avenues for treatment. For example, RNA-based therapies could be combined with smart drug delivery systems or tissue-engineered constructs to enhance their efficacy and precision. RNA therapeutics stand at the forefront of a medical revolution, offering the potential to provide more precise, personalized, and effective treatments for a wide range of diseases. As research progresses and technical barriers are overcome, we can envision a future where RNA-based drugs play a central role in healthcare, addressing previously untreatable conditions and fundamentally changing our approach to disease. The coming years are likely to see an explosion in the applications of RNA therapeutics, ushering in a new era of medicine and patient care that promises to redefine our understanding and treatment of human diseases.

The field of RNA therapeutics stands at the cusp of a revolutionary transformation in medicine, with immense potential to address a wide range of diseases through precise, personalized approaches. As we look to the future, several key areas are likely to drive significant advancements:

(1) Personalized Medicine Revolution: RNA therapeutics are uniquely positioned to spearhead the era of truly personalized medicine. The ability to rapidly design and manufacture RNA-based drugs tailored to an individual’s genetic profile will likely lead to more effective treatments for rare genetic disorders and complex diseases like cancer. We may see the emergence of hospital-based RNA therapeutic platforms capable of producing patient-specific treatments on demand.
(2) Advanced Delivery Systems: The development of next-generation delivery systems will be crucial in overcoming current limitations. We can expect to see innovations in lipid nanoparticle technology, biomimetic nanoparticles, and cell-based delivery systems. These advancements will likely improve tissue-specific targeting, reduce off-target effects, and enhance the overall efficacy of RNA therapeutics.
(3) Combination Therapies: The future will likely see an increase in combination therapies involving RNA drugs. This could include combinations of different RNA modalities (e.g., siRNA with miRNA) or RNA therapeutics paired with traditional small-molecule drugs. Such approaches may offer synergistic effects, particularly in complex diseases like cancer.
(4) Expanded Applications: While current RNA therapeutics focus primarily on genetic disorders and certain cancers, future applications are likely to expand into areas such as regenerative medicine, immunomodulation, and even aging-related conditions. The versatility of RNA as a therapeutic modality will open new avenues for treating previously “undruggable” targets.
(5) AI Integration: The integration of AI in RNA drug design and optimization will likely accelerate development timelines and improve efficacy. These technologies could help predict off-target effects, optimize delivery strategies, and even personalize treatment regimens.
(6) Regulatory Evolution: As RNA therapeutics become more prevalent, regulatory frameworks will need to evolve to accommodate their unique characteristics. We may see the development of specialized regulatory pathways for RNA-based drugs, particularly for personalized therapies.
(7) Manufacturing Innovations: Advancements in manufacturing technologies will be crucial to meet the growing demand for RNA therapeutics. This may include the development of more efficient and scalable production methods, as well as innovations in quality control and stability enhancement.
(8) RNA Editing Technologies: The emergence of RNA editing technologies, such as CRISPR-based systems targeting RNA, could open new possibilities for transient and reversible genetic modifications, further expanding the therapeutic potential of RNA-based approaches.

RNA therapeutics represent a revolutionary field in medicine with the potential to transform healthcare. However, three significant challenges remain for their widespread application. Addressing and overcoming these challenges will be crucial to unlocking the true potential of RNA therapeutics. The first challenge concerns the “delivery system” for delivering RNA drugs to specific tissues or cells. RNA molecules are relatively large and charged, making it difficult for them to pass through cell membranes and reach target tissues unaided. To address this issue, researchers are developing innovative approaches. For instance, advancements in delivery systems using liposomes (capsules made from phospholipids, which are components of cells and biological membranes) and nanoparticles are progressing. These systems can protect RNA molecules while efficiently delivering them to specific tissues or cells. Nanoparticles targeting cell-specific receptors are being developed, enabling more precise tissue-specific delivery. The second challenge involves “RNA stability”. RNA is inherently unstable and rapidly degraded by RNA-degrading enzymes in the body. To combat this, researchers are developing RNA chemical modification techniques. For example, introducing chemical modifications such as 2′-*O*-methyl modification, 2′-fluoro modification, and phosphorothioate bonds to RNA molecules can improve RNA stability and extend its half-life in the body. Additionally, the use of circular RNA (RNA chains with their ends joined to form a circle) is gaining attention. Circular RNA is more stable as it is not affected by RNA-degrading enzymes like linear RNA. The third challenge is the “off-target effect” (the phenomenon of affecting regions or genes other than the intended target). If RNA drugs influence the expression of unintended genes, unexpected side effects may occur. To address this, various approaches are being studied, including precise sequence design using bioinformatics tools and reduction of off-target effects through chemical modifications. Furthermore, high-precision RNA editing technology using the CRISPR–Cas13 system is being developed, which is expected to enable more specific control of gene expression. Continuous research and technological innovation in addressing these challenges are steadily expanding the possibilities of RNA therapeutics. The potential applications of RNA drugs extend beyond treatment to prevention and diagnosis. For instance, mRNA vaccines have shown remarkable efficacy in preventing infectious diseases, playing a central role in responding to the COVID-19 pandemic. This success has demonstrated the flexibility and rapid development cycle of RNA technology, paving the way for the development of preventive vaccines against other infectious diseases and cancers. RNA aptamers are also promising as diagnostic tools. With their ability to bind to specific molecules with high affinity and specificity, RNA aptamers are expected to be applied as new biosensors and molecular imaging probes. Moreover, the development of “aptamer–nanoparticle complexes”, combining RNA aptamers and nanoparticles, is being considered for high-sensitivity detection of cancer cells [94]. As research progresses, more RNA therapeutics are entering clinical stages. RNA drugs are bringing new hope to patients with previously untreatable diseases and have the potential to fundamentally change treatment approaches. The continuous evolution of RNA therapeutics such as ASOs, RNAi, and RNA aptamers is expanding the range of targetable diseases and pushing the boundaries of modern medicine [95]. The future of RNA therapeutics will become even brighter by overcoming these challenges and continuing technological innovation. RNA therapeutics hold the potential to significantly change the future of medicine, including the realization of personalized medicine and providing new approaches to diseases that have been difficult to treat. Great expectations are placed on the progress of future research and development in this field [5].

In conclusion, the future of RNA therapeutics is bright and filled with potential. Furthermore, there is also an intriguing target called long non-coding RNA [96]. As these technologies continue to mature, we can anticipate a paradigm shift in how we approach disease treatment and prevention, moving towards more precise, personalized, and effective therapeutic strategies. The coming years will likely witness an explosion of RNA therapeutic applications, marking a new era in medical science and patient care.

## Figures and Tables

**Table 1:** Types of RNA therapeutics and their applications.

RNA Therapeutic Type	Mechanism	Key Applications	API Example
Antisense oligonucleotides (ASOs)	Modulate gene expression through complementary binding	Neurodegenerative diseases, genetic disorders	Nusinersen (Spinraza), inotersen (Tegsedi), golodirsen (Vyondys 53)
Small interfering RNAs (siRNAs)	Gene silencing through RNA interference	Cancer, genetic disorders, cardiovascular diseases	Patisiran (Onpattro), givosiran (Givlaari), inclisiran (Leqvio)
MicroRNAs (miRNAs)	Regulate gene networks	Cancer, cardiovascular diseases	Cobomarsen (MRG-106)
Messenger RNAs (mRNAs)	Protein replacement therapy	Genetic disorders, infectious diseases (vaccines)	Moderna and Pfizer-BioNTech COVID-19 vaccines
Aptamers	Bind specific targets with high affinity	Diagnostic tools, targeted drug delivery	Pegaptanib (Macugen)
CRISPR–Cas9 guide RNAs	Direct genome editing	Genetic disorders, cancer	Alt-R CRISPR–Cas9 System, CRISPR Therapeutics’ CTX001

**Table 2:** The advantages and disadvantages of each type of drug delivery system.

Delivery System	Advantages	Disadvantages	Examples of Marked APIs
Lipid Nanoparticles (LNPs)	Efficient encapsulation of RNA, enhanced cellular uptake, improved stability, versatile and tunable, proven success in clinical applications	Limited biodistribution beyond liver, potential immunogenicity, manufacturing and quality control challenges, storage and transportation stability issues	Moderna and Pfizer-BioNTech COVID-19 vaccines (mRNA), patisiran (Onpattro) for hereditary, transthyretin-mediated amyloidosis (siRNA)
Polymeric Nanoparticles (PNPs)	High tailoring ability, biodegradability, ease of functionalization, good drug release profile, potential for stimuli-responsive delivery	Potential toxicity of cationic polymers, challenges in large-scale production, complex polymer–RNA interactions	No FDA-approved RNA therapeutics yet, several candidates in clinical trials
Conjugation Strategies	Enhanced stability, improved cellular uptake, targeted delivery, reduced immunogenicity	Limited to specific tissues/cell types, potential alteration of RNA activity, complex synthesis and characterization	Givosiran (Givlaari) for acute hepatic porphyria (siRNA–GalNAc conjugate), inclisiran (Leqvio) for hypercholesterolemia (siRNA–GalNAc conjugate)
Viral Vectors	High transduction efficiency, long-term gene expression (for some vectors), tissue-specific targeting	Immunogenicity concerns, limited payload capacity, potential for insertional mutagenesis	No FDA-approved RNA therapeutics yet, several candidates in clinical trials for gene therapy

**Table 3:** Clinical applications and approved RNA drugs.

Disease	RNA Therapeutic Approaches	Examples/Progress	Challenges/Prospects
Neurodegenerative Diseases	ASOs, RNAi	Promising clinical trials for spinal muscular atrophy and Huntington’s disease	Delivery across blood–brain barrier, widespread distribution in CNS
Genetic Disorders	ASOs, RNAi, mRNA therapy	Patisiran (Onpattro) and vutrisiran (Amvuttra), mRNA therapy for metabolic disorders	Potential to treat a wide range of genetic conditions
Cancer Treatment	mRNA vaccines, ASOs. RNAi, mRNA therapy	Tumor-specific antigen-encoding mRNA vaccines, silencing oncogenes, restoring tumor suppressor genes	Combination with existing treatments, overcoming drug resistance
Infectious Diseases	mRNA vaccines, RNAi, ASOs, RNA aptamers	COVID-19 mRNA vaccines, siRNAs targeting viral genes	Rapid response to emerging threats, potential solutions for antibiotic-resistant bacteria
Cardiovascular Diseases	RNAi, ASOs, mRNA therapy, microRNA therapeutics	siRNAs for hypertension and LDL cholesterol management, VEGF mRNA therapy for myocardial revascularization	Targeting lipid metabolism genes, potential treatments for atherosclerosis

**Table 4:** Challenges and strategies in RNA therapeutics.

Challenge	Description	Strategies to Overcome
Stability	RNA degradation in biological fluids	Chemical modifications, nanoparticle encapsulation
Off-target effects	Unintended gene modulation	Careful sequence design, chemical modifications
Immunogenicity	Immune system activation	Modified nucleosides, optimized delivery systems
Delivery to target tissues	Difficulty reaching specific organs/cells	Lipid nanoparticles, conjugation strategies, targeted delivery

## Data Availability

No new data were created.
